# Xerodermia and Allied Affections

**Published:** 1907-07-27

**Authors:** 


### Dermatology.

### XERODERMIA AND ALLIED AFFECTIONS.

There are many grades of roughness and dryness of the skin. The mildest degree of all is really nothing more than a " normal abnormality," and it seldom calls for special treatment. Nevertheless it is the harsh, lack-lustre condition of the integument which is particularly liable to a squamous or a fissured eczema. Another form of roughness is that due to prominence with actual hypertrophy of the pilo-sebaceous follicles which is usually most marked upon the outer aspects of the limbs, and is known as keratosis pilaris. This may be described as an exaggerated and permanent condition of " goose-flesh," often found in individuals who have no other trace of any cutaneous affection. Sometimes there is, in addition, a fine desquamation of the surface which gives to the skin an appearance as if it had been lightly powdered with dust. In other cases the normal cleavage-lines of the epidermis are intensified and the skin has a dirty, unwashed look about it; in fact, patients will volunteer the statement that it is most difficult to get their skins properly clean, for they seem to " hold the dirt." Ichthyosis, as the name implies, is the more advanced condition of xerodermia in which the skin is covered with adherent, overlapping scales like those of a fish. It is seen to perfection upon the integument of the shins, and the legs are the parts to examine when it is suspected that some degree of ichthyosis exists. Xerodermia and its congeners may be grouped among the family diseases—*i.e.*, they are congenital in origin and tend to be transmitted through successive generations.

The aim and object of treatment is to render the skin once more smooth and supple by polishing down all rough and scaly projections of the surface and by stimulating the secretions of the sweat and sebaceous glands. Mild cases respond very well to frequent warm baths which may contain a little sodium bicarbonate, one drachm to a gallon of water. After drying, the surface should be well anointed with lanoline or a mixture of equal parts of lanoline and vaseline containing from 5 to 10 grains of salicylic acid to the ounce, according to the degree of scaliness present. The official ointment of the glycerine of the subacetate of lead has also been found very useful, while the glycerine of starch, diluted with warm water, is another excellent application. In more obstinate cases the addition of a little resorcin (5 gr. ad 1 oz.) or ichthyol (3 to 8 gr.) with an increased quantity of salicylic acid may be tried with benefit. Cod-liver oil internally, especially in poorly nourished children, seems to help external medication wonderfully. Young adults may take small doses of thyroid extract, beginning with 2½ grains twice a day. This drug should never be administered when regular and frequent medical inspection of the case is impracticable. The condition is apt to relapse if treatment be discontinued too soon, especially in cold weather.

